# Supplementary material for: “Food Is Our Love Language”: Using Talanoa to Conceptualize Food Security for the Māori and Pasifika Diaspora in South-East Queensland, Australia
Source: Nutrients. 2022 May 11;14(10):2020. doi: 10.3390/nu14102020 (PMC9143296; doi:10.3390/nu14102020)
Supplement: Supplementary file 1 [file nutrients-14-02020-s001.zip › nutrients-1700995-supplementary.pdf]

S1. SQRQ* Checklist for Qualitative Research [81].

http://www.equator-network.org/reporting-guidelines/srqr/ (accessed on 11 April 2022)	Page/line no(s).
**Title and abstract**	
**Title**—Concise description of the nature and topic of the study identifying the study as qualitative or indicating the approach (e.g., ethnography, grounded theory) or data collection methods (e.g., interview, focus group) is recommended	p 1
**Abstract**—Summary of key elements of the study using the abstract format of the intended publication; typically includes background, purpose, methods, results, and conclusions	p 1 13–24
**Introduction**	
**Problem formulation**—Description and significance of the problem/phenomenon studied; review of relevant theory and empirical work; problem statement	Food security defined and determinants<br>p 1<br>Significance of problem for MP<br>P2
**Purpose or research questio**n—Purpose of the study and specific objectives or questions	P3 lines 105–109
**Methods**	
**Qualitative approach and research paradigm**—Qualitative approach (e.g., ethnography, grounded theory, case study, phenomenology, narrative research) and guiding theory if appropriate; identifying the research paradigm (e.g., postpositivist, constructivist/ interpretivist) is also recommended; rationale**	Talanoa described PAR described p 3 lines 113–132
**Researcher characteristics and reflexivity—**Researchers' characteristics that may influence the research, including personal attributes, qualifications/experience, relationship with participants, assumptions, and/or presuppositions; potential or actual interaction between researchers' characteristics and the research questions, approach, methods, results, and/or transferability	Section 2.5 p 7 lines 231–241
**Context**—Setting/site and salient contextual factors; rationale**	This is provided in introduction p 1 Lines 61–102
**Sampling strategy**—How and why research participants, documents, or events were selected; criteria for deciding when no further sampling was necessary (e.g., sampling saturation); rationale**	Section 2.2 p 3 lines 136–151
**Ethical issues pertaining to human subjects—**Documentation of approval by an appropriate ethics review board and participant consent, or explanation for lack thereof; other confidentiality and data security issues	Section 2.6 p 7 lines 243–253
**Data collection methods**—Types of data collected; details of data collection procedures including (as appropriate) start and stop dates of data collection and analysis, iterative process, triangulation of sources/methods, and modification of procedures in response to evolving study findings; rationale**	Section 2.3 p 4 lines 153–172
**Data collection instruments and technologies**—Description of instruments (e.g., interview guides, questionnaires) and devices (e.g., audio recorders) used for data collection; if/how the instrument(s) changed over the course of the study	Section 2.3 p 4 lines 153–172
**Units of study**—Number and relevant characteristics of participants, documents, or events included in the study; level of participation (could be reported in results)	Table 1 p 8 and p 7 lines 256–263
**Data processing**—Methods for processing data prior to and during analysis, including transcription, data entry, data management and security, verification of data integrity, data coding, and anonymization/de-identification of excerpts	Section 2.3 p 4

**Data analysis**—Process by which inferences, themes, etc., were identified and developed, including the researchers involved in data analysis; usually references a specific paradigm or approach; rationale**	Section 2.4 pp 5–7
**Techniques to enhance trustworthiness**—Techniques to enhance trustworthiness and credibility of data analysis (e.g., member checking, audit trail, triangulation); rationale**	pp 196–220
**Results/findings**	
**Synthesis and interpretation**—Main findings (e.g., interpretations, inferences, and themes); might include development of a theory or model, or integration with prior research or theory	Themes identified and summarized in Figure 3
**Links to empirical data—**Evidence (e.g., quotes, field notes, text excerpts, photographs) to substantiate analytic findings	Each theme is accompanied with quotes pp 9–18
**Discussion**	
**Integration with prior work, implications, transferability, and contribution(s) to the field—**Short summary of main findings; explanation of how findings and conclusions connect to, support, elaborate on, or challenge conclusions of earlier scholarship; discussion of scope of application/generalizability; identification of unique contribution(s) to scholarship in a discipline or field	Short summary lines 662–673<br>Discussion of broader implication pp 17–20
**Limitations**—Trustworthiness and limitations of findings	Section 4.4 lines 804–819
**Other**	
**Conflicts of interest**—Potential sources of influence or perceived influence on study conduct and conclusions; how these were managed	Page 21 lines 872–878
**Funding**—Sources of funding and other support; role of funders in data collection, interpretation, and reporting	Page 21 lines 852, 878–880

*The authors created the SRQR by searching the literature to identify guidelines, reporting standards, and critical appraisal criteria for qualitative research; reviewing the reference lists of retrieved sources; and contacting experts to gain feedback. The SRQR aims to improve the transparency of all aspects of qualitative research by providing clear standards for reporting qualitative research. **The rationale should briefly discuss the justification for choosing that theory, approach, method, or technique rather than other options available, the assumptions and limitations implicit in those choices, and how those choices influence study conclusions and transferability. As appropriate, the rationale for several items might be discussed together. SRQR, Standards for Reporting Qualitative Research; MP, Māori and Pasifika; PAR, Participatory Action Research.

S2. Community Researchers (CR) interview guide.

Introduction

Peer researchers will introduce themselves (see example of script):

- Where you are from and the community you belong with
- Explain the aims and purpose of the research project.
- Explain clearly that the research is about the wider community and that individuals questioned are not thought to be inherently food insecure
- Discuss the consent and what participants agree/disagree with anonymity, privacy, confidentiality—and if you wish to be personally acknowledged in the reports and presentations with appropriate consent given.
- Please note same questions asked below can be answered by the youth researchers where they may like to relate their narratives as they talanoa with the participants!

About the participants

Tell me a little bit about yourself?

- *How do you identify yourself (Probe: where you come from, what community do you belong to, what is your cultural/ ethnic background, are you 1st, 2nd or 3rd generation Maori & Pacific Islander etc.)?*
- *For first generation: Tell me about your journey to Australia?*
- *Second or more generation: How well are you connected to your community?*
- *What's your role in your community?*

Food and culture in the community and family/household. These questions ask why food is important in a particular context such as social gathering or events, in your family or community:

1. Food and culture

What is the cultural connection between food and your culture or community identity? *How important is this and why?*

- *Can you give some examples such as Island or traditional foods is important to your cultural identity or has important community significance?*
- *How are these foods important in terms of space, spirituality/religion, family relationships & connections, hospitality, feeling safe, wellbeing?*

Do you think there is any community expectations (obligations) around providing food during a service, events or social functions? *What are these expectations and why are they important in your community?*

Community context

How is the use of food expressed within your <______> community? Why?

- *In what situations is food used? (Can you give some examples of situations such as family meetings, celebrations, and community events where specific types of food/or food is important)*
- *How would members of your community feel if they couldn't contribute food in such situations? (or if they talk from their own experience—then how would you feel if you couldn't contribute food in such situations?)*

How important is giving and receiving gifts of food (reciprocity) when participating participation in community/social life as you have described above?

Is the way of sharing food changing in your community? How is this changing and why?

In your community, are you aware of any situations/instances where providing or being expected to provide food may cause hardship?

What do families do If they are unable to meet the expectations where they are unable contribute or participate in social occasions?

Family/household context

What do you think is the general situation with food within families/ or households in your community?

- *Explain how food is prepared and eaten generally within families/households in your community? Who usually does the shopping? Who makes the decisions about what is prepared and eaten? Who usually does the cooking—is this shared responsibility e.g., mum, dad, kids, grandparents etc.? Is this experience similar to your family/household?)*

What prevents families/households from making these changes or getting what they want to eat? Why*?*

Note: *If they are unable to talk about the community bring it back to their household!*

2. Food Insecurity and support available

Are there any challenges or difficulties with putting food on the table for Maori & Pasifika families? *What are these challenges or difficulties? Why do you think it is difficult? There may indicate there are no challenges so you can ask about support for families who have difficulties putting food on the table—next series of questions!*

How do you think families feel if they cannot put food on the table?

How do you or your community support each other, find solutions or ways to ensure food is available to your families? *(Can you give some examples)*

- *When you have family gatherings, how do you help each other?*
- *What resourceful ways to people provide enough food / access food?*

3. Solutions or ways to support food security

What do you think needs to happen to help families/households to put food on the table?

What more can government, community or church organisations do to ensure food security for your community/ family?

What types of solutions or support do your community/family need?

Can you give examples of what support may look like?

What solutions do you think should be available and useful to your community?

Is there anything else that you think may be useful to this project that we haven't covered?

S3. Contact Summary Sheet (CSS) template used in data analysis [49].

**Interview code**
**Date of interview**
**Interviewer:**
**Author of this summary**
**What were the main issues or themes that struck you with this interview?**
**Anything that struck you as interesting, important or touched your heart as you listen to this interview?**
**What were key take home messages about the importance and role of food in the participant's culture, at a community level or at a household level?**
**What were key take home messages about the concept of food security or insecurity?**
**What were key take home messages about support available, strategies or potential solutions?**
**Other Quotes**
**Background**

S4. List of Steering Committee (SC) and Community Researchers (CR).

Steering Committee				
**Cultural Identification**	**Elder (E)/Community Member (CM)**	**Male/Female**	**Steering Committee Tenure**	**Community Researcher**
Samoan	CM	F	2019	X
Fijian	E	F	2019–present	✓
Māori	E	F	2019–present	✓
Samoan	CM	M	2019	X
Tongan/Australian	CM	F	2019–present	✓
FSM/Solomon Islander	CM	F	2019–present	✓
Papua New Guinean	E	F	2019–present	✓
Rotuman	CM	F	2019–present	✓
Samoan	CM	F	2019–present	✓
Samoan	E	F	2019–present	✓
Niuean	CM	M	2021	X
Fijian	CM (Lead Researcher)	F	2019–present	✓
Australian	University Researcher	F	2019–present	X
**Additional Community Researchers**				
**Cultural Identification**	**Older (O) or Young (Y)**	**Male/ Female**		
Aboriginal/Fijian	Y	F		
Papua New Guinean	O	M		
Māori/Australian	Y	M		
Samoan	Y	F		
Solomon Islander/I-Kiribati	Y	M		

X, absent; ✓, present.

S5. Exemplar quotes for identity sub-themes.

**Sub-theme**	**Exemplar quote**
Food as connection	*So culturally, food for me is the ongoing connection. Not only to me, with my culture, but to me who I'm eating with. And that connection is a really valued piece of my identity. When we eat well, we prosper, we are you know more productive, we have a better life (O_ Māori).*
Food as a language of love	*It's like, you know, it's sort of, like a love language in ways you know what I mean giving and receiving food. Younger people like myself and yourself. I don't think we realize the value that is placed on food. (Y_ Papua New Guinea).*
Food as a way of honouring family and showing respect	*I think it's really important to, it's just a common practice and a very important thing to pay your respects. If I'm entering into someone's house, if we are going somewhere, it's always a practice to express our respect and our appreciation for us coming into your space. And so, it has this connotation to it. We are very respectful of our entrance into other people's space, the other, you know, what is their belongings and things… so it can be with food or other cultural elements... It is always just that appreciation and respect, and humility (M_ Tonga).*
Maintaining a system of hierarchy and status	*Certain foods are shared in social occasions or functions and have higher value such as ufi (dalo) and so these are shared or gifted with families and members as a sign of respect… For instance, different parts of fish or meat are given to certain important members of the family (usually the head or the eldest (O_Fiji).* *I think a lot of Pacific people understand that food comes with status. You know, like, it may not be said, but like, if you go to an event, and the food is bad, then whoever organized that event, their status kind of automatically drops. (Y_ Samoa).*
Traditional foods and cultural identity - 'keeping culture alive'	*We had one (underground oven) at the premises and one again at campsite. We have a number of youths [who] turned up and you can tell they thoroughly enjoyed the experience. This is how we cook back at home, how our ancestors cooked. It wasn't with an electric oven that we turn the thing on. It was with wood and rocks. (O_ Niue).*
Creating culturally safe places	*It's Umu Kitchen] a safe place. Just like what we've done with our restaurant... It's a safe place, physically, mentally, spiritually and financially. You don't have to be rich to afford to come. If you have nothing, you still can come. Give me something. It's like, the culture and like in Tonga… someone make... (Ulu') and everybody share (M_ Tonga).*
